# Metal ion homeostasis regulates condensin-dependent chromatin architecture and chromosome segregation in *Schizosaccharomyces pombe*

**DOI:** 10.71150/jm.2505008

**Published:** 2025-08-29

**Authors:** Seong Ho An, Kyoung-Dong Kim

**Affiliations:** Department of Systems Biotechnology, Chung-Ang University, Anseong 17546, Republic of Korea

**Keywords:** condensin, metal ion homeostasis, genome organization, *Schizosaccharomyces pombe*

## Abstract

Condensin plays a central role in mitotic chromosome organization and segregation by mediating long-range chromatin interactions. However, the extent to which cellular metabolic status influences condensin function remains unclear. To gain insights into the relationship of metal ion homeostasis and the function of condensin, we conducted genome-wide chromatin immunoprecipitation sequencing (ChIP-seq) using *Schizosaccharomyces pombe* under iron- or zinc-deficient conditions. Under iron- or zinc-deficient conditions, ChIP-seq results revealed a selective reduction in condensin binding at high-affinity target loci, particularly genes regulated by Ace2 and Ams2, while cohesin binding remained largely unaffected. Hi-C analysis showed that iron depletion weakened chromatin interactions at these condensin targets and centromeres, without disrupting global genome architecture. DNA fluorescence in situ hybridization (FISH) confirmed that iron deficiency impaired long-range associations between centromeres and Ace2 target loci at the single-cell level. Notably, iron deficiency led to chromosome segregation defects during mitosis, suggesting that diminished condensin occupancy compromised genome stability. These changes occurred without significant alterations in condensin protein levels or global transcription, indicating a direct effect of metal ion availability on condensin activity. Collectively, our findings revealed a previously unrecognized regulatory axis in which cellular metal ion homeostasis modulated condensin-dependent chromatin organization and mitotic chromosome segregation, offering new insights into the integration of metabolic state with genome maintenance.

## Introduction

The structural maintenance of chromosomes (SMC) complexes, cohesin and condensin, are essential regulators of genome organization and stability. Cohesin mediates sister chromatid cohesion, shapes interphase chromatin structure, and modulates gene expression (Nasmyth and Haering, 2009). In contrast, condensin is primarily active during mitosis, where it compacts chromosomes and ensures their accurate segregation (Hirano, 2012). Although these complexes share structural features and partially colocalize at genomic elements, such as tRNA genes and LTR retrotransposons (Kim et al., 2016b), they exhibit distinct binding preferences. Cohesin is enriched at convergent intergenic regions and maintains stable chromatin domains throughout the cell cycle (Lengronne et al., 2004; Mizuguchi et al., 2014), whereas condensin preferentially binds histone genes and mitotically induced genes regulated by Ace2 and Ams2 (Kim et al., 2016b; Sutani et al., 2015). Importantly, condensin-mediated chromatin interactions are established during mitosis and disassembled thereafter (Kakui et al., 2017; Tanizawa et al., 2017).

In *Schizosaccharomyces pombe*, condensin organizes mitotic chromosomes by facilitating long-range chromatin interactions, including centromeric clustering and associations between centromeres and Ace2/Ams2 target loci (Iwasaki et al., 2010; Kim et al., 2016b). Loss of condensin disrupts these interactions and leads to chromosome missegregation, underscoring its critical role in mitotic genome architecture (Nakazawa et al., 2019; Ono et al., 2004).

Beyond cell cycle regulation, increasing evidence indicates that three-dimensional genome organization responds dynamically to environmental and metabolic cues. In both yeast and metazoans, chromatin architecture is remodeled under stress and nutrient fluctuations. For instance, heat shock triggers genome reorganization via transcriptional condensates in *S. cerevisiae* (Chowdhary et al., 2022) and relocalization of architectural proteins in *Drosophila* (Li et al., 2015). In mammals, dietary composition influences higher-order chromatin interactions, as seen in response to high-fat or carbohydrate-rich diets (Qin et al., 2020). Nevertheless, the impact of essential metabolic cofactors, such as metal ions on chromatin structure and chromosome dynamics, remains poorly characterized.

Iron and zinc are indispensable metal cofactors that contribute to DNA metabolism and chromatin regulation. In *S. pombe*, iron homeostasis is governed by Fep1, a transcriptional repressor of iron-responsive genes (Labbé et al., 2007; Pelletier et al., 2005), while zinc levels are regulated by Loz1, which represses target genes under zinc-replete conditions (Corkins et al., 2013; Wilson et al., 2019). Iron is required for DNA replication, repair, and the activity of histone-modifying enzymes (Muckenthaler et al., 2017), whereas zinc supports chromatin accessibility and stabilizes numerous transcription factors (Klug, 2010; Ocampo et al., 2024). Notably, iron homeostasis has been linked to facultative heterochromatin regulation and environmentally responsive gene expression (Gallagher et al., 2018). Moreover, previous work has identified transcriptional upregulation of iron-uptake genes in the *cut14-208* condensin mutant (Tanizawa et al., 2017). Although connections between metal availability and chromatin accessibility have been reported, their direct influence on condensin-dependent mitotic genome organization and chromosome segregation remains unclear.

In this study, we observed same upregulation of iron-uptake genes upon acute condensin depletion using an auxin-inducible degron (AID) system and investigated how iron and zinc deficiency influence SMC complex function, genome organization, and chromosome segregation in *Schizosaccharomyces pombe*. Intriguingly, iron deficiency alone also induced striking chromosome segregation defects, raising the possibility that metal availability may directly modulate condensin activity. Specifically, we analyzed the binding patterns of condensin and cohesin, as well as condensin-mediated chromatin interactions, under metal ion-deficient conditions.

## Materials and Methods

### *S. pombe* strains and cell culture

All *S. pombe* strains used in this study are listed in Table S1. Strain FY9897 was used for the temperature-sensitive (ts) *cut14-208* mutant, while FY14415 was used for the mini-chromosome loss assay. The *cut14* and *cut3* genes were C-terminally tagged with the auxin-inducible degron peptide 5-Ada-IAA and integrated into strain FY39926, which expresses *Padh1-OsTIR1-F74A*, as described previously (Zhang et al., 2022). Strains FY9897, FY14415, and FY39926 were obtained from the National Bio-Resource Project (Japan Agency for Medical Research and Development, Japan). Epitope tags (Pk or Myc) were added to the C termini of Cut14 (condensin), Cnd2 (condensin), and Rad21 (cohesin) using a PCR-based module method (Bähler et al., 1998).

All strains were cultured in yeast extract supplemented (YES) medium. The *cut14-208* ts mutant was cultured at 26°C and shifted to 36°C for 1 h. To deplete condensin subunits (Cut14-3sAID and Cut3-3sAID), 100 nM 5-adamantyl-IAA (TCI, A3390) was added to the YES medium. For iron repletion and depletion, 100 µM ferric chloride (Sigma, F2877) and 250 µM 2,2′-bipyridyl (Sigma, D216305) were added to the YES medium and incubated at 30°C for 90 min. For zinc depletion, TPEN (N,N,N′,N′-tetrakis(2-pyridylmethyl)ethylenediamine; Sigma, P4413) was added to a final concentration of 50 or 100 µM and incubated at 30°C for 2 h.

### DAPI-staining after HU arrest

Mid-log phase cells were cultured in YES medium containing 11 mM hydroxyurea (HU) at 30°C for 4 h and then released into fresh YES medium containing 250 µM 2,2′-bipyridyl for iron depletion. Following cell-cycle synchronization, nuclei were stained with 4′,6-diamidino-2-phenylindole (DAPI). At least 200 nuclei were counted per time point.

### Mini-chromosome loss assay

The mini-chromosome loss assay was performed as described previously (Allshire et al., 1995; Niwa, 2018), with minor modifications. Mid-log phase cells were treated with 250 µM 2,2′-bipyridyl for 2 h in YES medium and plated on YE medium containing low adenine (12 mg/L). Plates were incubated at 30°C for 3 d and at 4°C for 1 d to enhance red pigmentation. More than 900 colonies were counted per experiment.

### Chromatin immunoprecipitation (ChIP)-seq and data analysis

ChIP was performed as previously described (Gadaleta et al., 2015), with modifications. Cells were cross-linked with 3% paraformaldehyde, followed by treatment with 10 mM dimethyl adipimidate. Chromatin was extracted using ChIP lysis buffer (50 mM HEPES–KOH, pH 7.5; 140 mM NaCl; 1 mM EDTA, pH 8.0; 1% Triton X-100; 0.1% sodium deoxycholate; 0.1 mM PMSF) supplemented with protease inhibitors (cOmplete, Roche, 11836153001) and lysed by bead beating. Lysates were sonicated using a Bioruptor (Diagenode) and precleared with protein G Dynabeads (Thermo, 10004D) at 4°C for 1 h. Immunoprecipitation was performed using anti-V5 tag antibody (Bio-Rad, MCA1360) or anti-Myc monoclonal antibody (Clontech, 631206). DNA was washed twice with ChIP lysis buffer, once with lysis buffer containing 650 mM NaCl, once with wash buffer (10 mM Tris-HCl, pH 8.0; 250 mM LiCl; 0.5% NP-40; 0.5% sodium deoxycholate; 1 mM EDTA), and once with TE buffer. Bead-bound DNA was eluted in TE containing 1% SDS at 65°C for 15 min with shaking (1000 rpm, repeated twice), followed by overnight incubation at 65°C and proteinase K digestion at 37°C for 5 h. DNA was purified using the QIAquick PCR Purification Kit (QIAGEN, 28104). Sequencing libraries were prepared using the NEBNext Ultra II DNA Library Prep Kit for Illumina (NEB, E7645) and NEBNext Multiplex Oligos for Illumina (NEB, E7335, Index Primers Set 1), with amplification using Q5 Master Mix (NEB, M0544). DNA fragments (200–700 bp) were size-selected using AMPure XP beads (Beckman Coulter, A63881) and sequenced on the Illumina NovaSeq platform with 100 bp paired-end reads. Reads were aligned to the *S. pombe* genome using Bowtie2 (v2.2.5), and peaks were visualized in TDF format using IGV tools. For quantitative analyses, RPKM values were calculated from a 1000-bp region surrounding the transcription start site (TSS) for Rad21-Myc and RNA polymerase II ChIP-seq and from a 250 bp region downstream of the transcription end site (TES) for Cut14-Pk. Real-time qPCR was performed using the CFX Connect Real-Time PCR Detection System.

### RNA extraction and cDNA synthesis

Total RNA was extracted using the RNeasy Mini Kit (QIAGEN, 74104) following the manufacturer’s instructions. Genomic DNA was removed using the TURBO DNA-free Kit (Thermo, AM1907). Complementary DNA (cDNA) was synthesized using RevertAid Reverse Transcriptase (Thermo, EP0442) with random hexamer primers. Transcript levels were quantified via real-time quantitative PCR (qPCR) using HOT FIREPol EvaGreen qPCR Mix Plus (Solis BioDyne, 08-24-00001). Relative expression was calculated using the ΔΔCt method, with *act1* as the reference gene.

### Western blotting

Mid-log phase cells were lysed in 0.1 M potassium phosphate buffer supplemented with protease inhibitors (PI) using bead beating. Protein concentrations were quantified using the Qubit Protein Assay Kit (Thermo, A50669) and normalized prior to loading. Proteins were resolved via SDS-PAGE on 8% polyacrylamide gels and transferred to nitrocellulose membranes (GE Healthcare, 10600004). Membranes were blocked with Smart-BlockTM Fast Blocking Buffer (Biomax, BWB-0500). Cut14-Pk and Cnd2-Pk were detected using a mouse anti-V5 tag antibody (Bio-Rad, MCA1360), followed by a goat anti-mouse IgG HRP-conjugated secondary antibody (Bio-Rad, 170-6516). β-tubulin was detected using a rabbit polyclonal anti-β-tubulin antibody specific for *S. pombe* (BioAcademia, 63-160), followed by a goat anti-rabbit IgG HRP-conjugated secondary antibody (Bio-Rad, 170-6515). Chemiluminescent signals were detected using WestGlowTM ECL reagents (Biomax, BWE0400), and images were acquired using an e-blot imager.

### Hi-C

Hi-C was performed as described previously (Tanizawa et al., 2017) with modifications. Cells were fixed in 3% paraformaldehyde for 10 min at room temperature, and cross-linking was quenched with 0.125 M glycine for 15 min. Fixed cells were lysed using FA lysis buffer and bead beating, followed by chromatin digestion with MboI (NEB, R0147S). Digested ends were filled in using biotin-14-dATP (Thermo, 19524-016), dCTP, dGTP, and dTTP with Klenow fragment (NEB, M0210), and ligated using T4 DNA ligase (NEB, M0202S). Cross-links were reversed by proteinase K treatment, and DNA was purified by phenol–chloroform extraction. Residual biotinylated nucleotides were removed using 1.8× AMPure XP beads (Beckman Coulter). Biotin-labeled DNA was sheared using a Bioruptor (Diagenode) and captured with MyOne Streptavidin C1 Dynabeads (Thermo, 65001). DNA was end-repaired and ligated to sequencing adaptors using the NEBNext Ultra II DNA Library Prep Kit (NEB). Bead-bound DNA was eluted by heating at 98°C for 10 min with shaking (900 rpm) and then amplified using NEBNext Multiplex Oligos for Illumina (Index Primers Set 1) and Q5 Master Mix (NEB) for 12 PCR cycles. DNA fragments (200–700 bp) were size-selected with AMPure XP beads and sequenced on the Illumina NovaSeq platform as 100 bp paired-end reads.

### Hi-C data analysis

Hi-C libraries were sequenced on an Illumina NovaSeq 6000 system as 100 bp paired-end reads. Reads were aligned to the *S. pombe* genome using the BWA-MEM algorithm (v0.7.17). All downstream Hi-C analyses were conducted using HiCExplorer (v3.7.5). Hi-C matrices with 10-kb resolution were generated using the hicBuildMatrix function. Matrix correction for read count bias was performed using hicCorrectMatrix, which removed bins with low or excessively high coverage. Normalization across samples was performed using hicNormalize, adjusting to the sample with the lowest read depth. Comparisons between normalized Hi-C matrices were made using the hicCompareMatrices function with the "log2ratio" option. Contact and differential interaction maps were visualized using hicPlotMatrix with the "clearMaskedBins" option.

### Fluorescence in situ hybridization (FISH)

FISH was conducted as described previously (Kim et al., 2016a). DNA probes targeting condensin-binding loci (*eng1* and *adg1*) and a control locus (*SPAC110*) were generated from ~16 kb PCR-amplified genomic DNA fragments. A centromeric probe was derived from plasmid pRS140 (Chikashige et al., 1989). DNA fragments and plasmid DNA were digested with 4-base cutters, purified by phenol extraction, and labeled with Cy3-dCTP or Cy5-dCTP (GE Healthcare, PA53021 and PA55021) using a random primer DNA labeling kit (TaKaRa, 6045). Images were captured using a Zeiss LSM800 Airy inverted confocal microscope equipped with oil immersion optics (Zeiss, Immersol). Z-stack images were acquired at 0.2 µm intervals using the ZEN Blue software.

## Results

### Condensin depletion led to transcriptional upregulation of iron-uptake genes

**Fig. 1.**
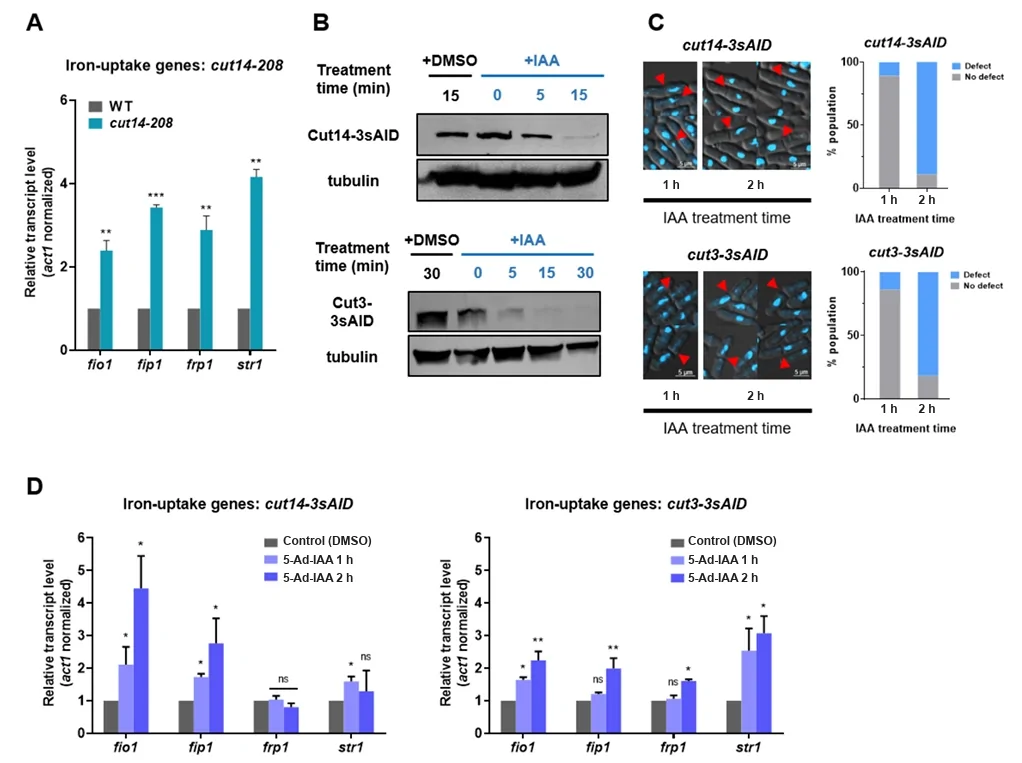
Misregulation of iron-uptake genes in the condensin mutant. (A) Transcript levels of iron-uptake genes in the condensin mutant (*cut14-208*). (B) Western blot showing depletion of Cut14 and Cut3 using the auxin-inducible degron (AID) system. 5-Adamantyl-IAA (100 nM) was used to induce degradation of AID-tagged proteins. (C) Chromosome segregation defects in the AID strain upon IAA treatment. The representative images show DAPI-stained cells exhibiting chromosome segregation defects following IAA treatment. Red arrows indicate cells with chromosomal segregation defects. More than 200 cells were analyzed. (D) Transcript levels of iron-uptake genes under condensin-depleted conditions using the AID system. Data represent the mean ± s.d. from three independent biological replicates and *p* values were calculated using a student’s t-test (^***^p < 0.001, ^**^p < 0.01, ^*^p < 0.05, ns: not significant).

A previous study has reported that iron-uptake genes, which are normally repressed by the transcription factor Fep1, are significantly upregulated in the *cut14-208* condensin mutant (Tanizawa et al., 2017). To validate this observation, we performed quantitative RT-PCR (qRT-PCR) and confirmed elevated expression of iron-uptake genes in *cut14-208* cells relative to wild-type controls (Fig. 1A). To rule out the possibility that this upregulation resulted from temperature shift, we generated AID strains for the condensin subunits Cut14 and Cut3 (Cut14-3sAID and Cut3-3sAID). Upon addition of 100 nM 5-adamantyl-IAA, Cut14-3sAID and Cut3-3sAID protein levels were rapidly depleted within 30 min, as confirmed by immunoblotting (Fig. 1B). Correspondingly, condensin depletion induced mitotic chromosome segregation defects (Fig. 1C), consistent with its essential role in mitosis. Furthermore, qRT-PCR analysis revealed a time-dependent upregulation of iron-uptake genes following auxin treatment in both *cut14-3sAID* and *cut3-3sAID* backgrounds (Fig. 1D), in agreement with the transcriptional changes observed in *cut14-208* condensin mutant (Fig. 1A). These findings indicate that condensin is required for the transcriptional repression of iron-uptake genes.

### Iron depletion impaired chromosome segregation fidelity

**Fig. 2.**
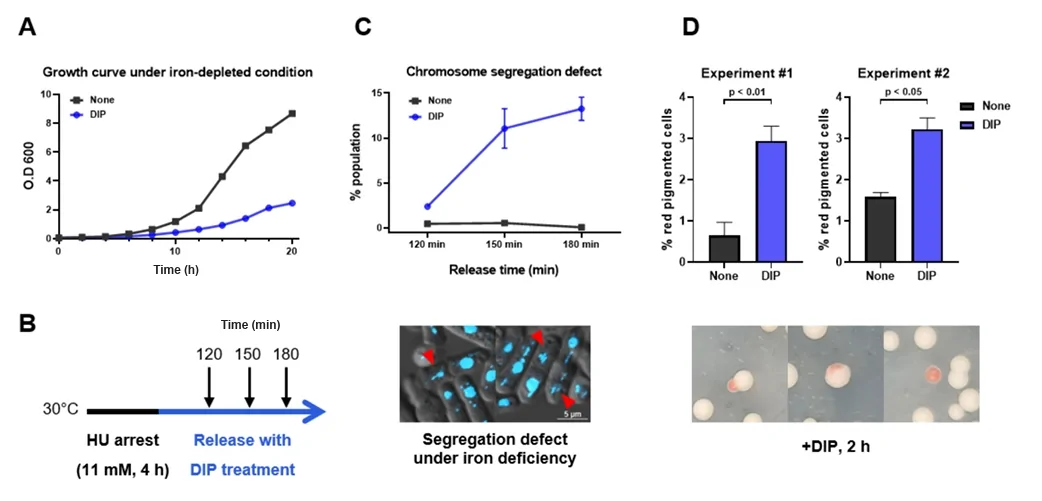
Effects of iron depletion on *S. pombe* growth and chromosome stability. (A) Growth curves of *S. pombe* in the presence or absence of 250 µM DIP in the YES medium. (B) Scheme of cell-cycle synchronization using hydroxyurea (HU). (C) Chromosome segregation defects under iron-depleted conditions. The representative images show DAPI-stained cells exhibiting chromosome segregation defects following DIP treatment. Red arrows indicate cells with chromosomal segregation defects. The percentage of cells with segregation defects was quantified at the indicated time points (upper panel). Red arrows indicate segregation defects (lower panel). (D) Effect of iron depletion on mini-chromosome stability. Experiments were repeated twice. Total cells counted: n = 1,146 and 1,287 (iron-replete); n = 966 and 1,105 (iron-depleted). Statistical validation was performed based on a Mann-Whitney U test.

Given the transcriptional up-regulation of iron-uptake genes in condensin-depleted cells, we next investigated whether iron availability itself affected condensin-dependent chromosome segregation. Cells grown under iron-depleted conditions exhibited significantly reduced growth, with final optical density (OD_600_) reaching ~2.0 compared to ~10.0 under normal conditions (Fig. 2A). To assess mitotic chromosome segregation, cells were synchronized in early S phase using HU and then released into iron-deficient medium by treatment with 2,2′-bipyridyl (DIP; Fig. 2B). Analysis of nuclear morphology revealed a marked increase in mitotic cells exhibiting phi-shaped chromosomes under iron-limiting conditions (Fig. 2C). To quantify chromosome segregation fidelity, we employed the Chr16 mini-chromosome loss assay. Iron-depleted cells displayed approximately a two-fold increase in mini-chromosome loss frequency relative to control cells (Fig. 2D). These results suggest that iron deficiency impairs faithful chromosome segregation during mitosis, although whether this reflects a direct effect on condensin function remains to be clarified.

### Iron depletion led to a global reduction in condensin binding, particularly at high-affinity target sites

**Fig. 3.**
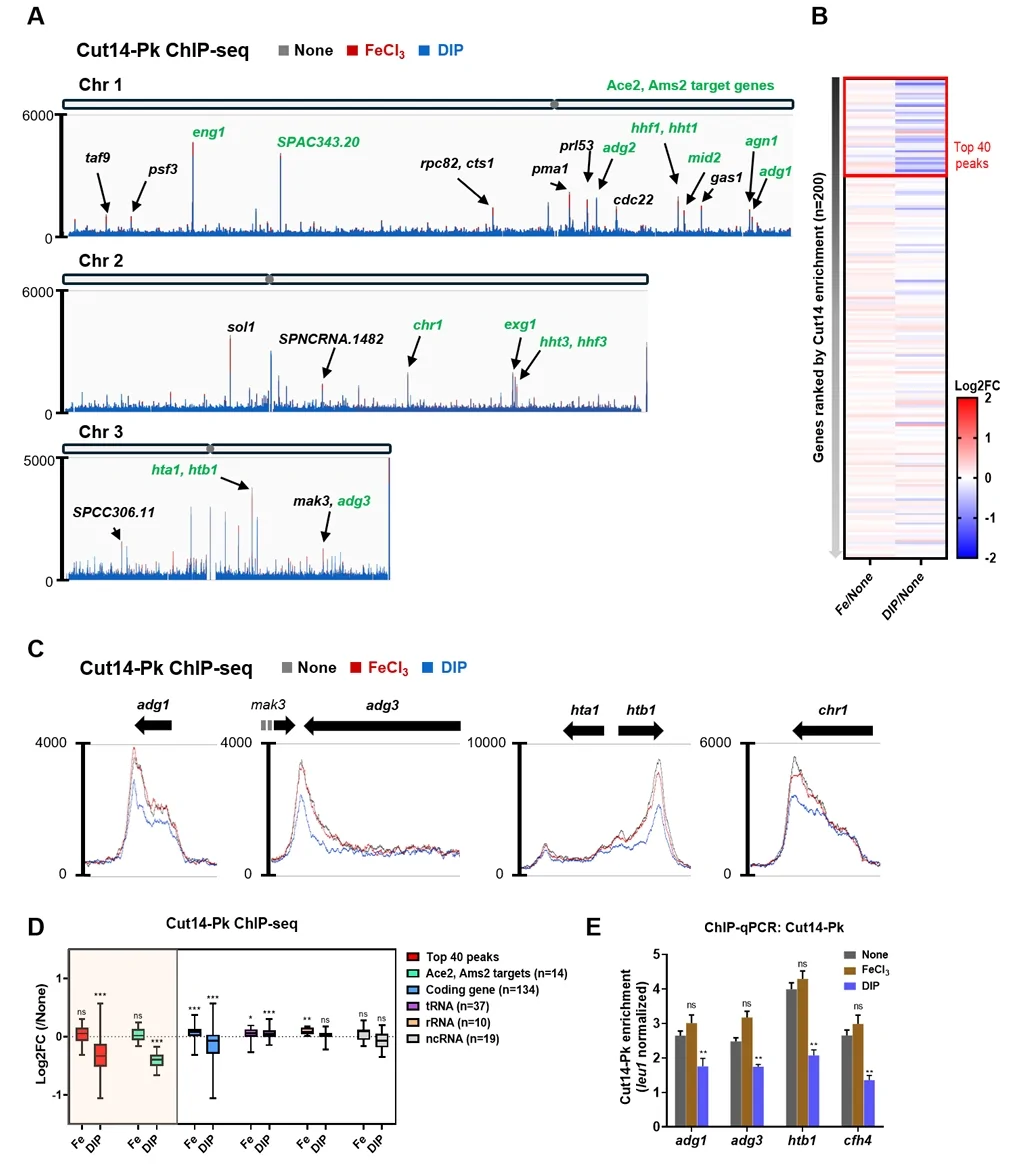
Reduced condensin binding under iron-depleted conditions. (A) ChIP-seq profiles of Cut14-Pk binding across chromosomes under iron-replete (100 µM FeCl_3_) and iron-depleted (250 µM DIP) conditions. Some genes among the top 200 condensin binding sites are shown in black, and Ace2/Ams2 target genes are shown in green. (B) Condensin binding profiles at the top 200 high-affinity sites under both conditions. (C) Decreased condensin binding at condensin target genes under iron-depleted conditions. Line plots show enrichment at target loci. Each snap is enlarged image captured from Integrative Genome Viewer (IGV). (D) Box plot showing average Cut14-Pk enrichment across different gene classes. *p* values were calculated using one sample t-test (^***^p < 0.001, ^**^p < 0.01, ^*^p < 0.05, ns: not significant). (E) ChIP-qPCR analysis of Cut14-Pk enrichment at Ace2/Ams2 targets (*adg1*, *adg3*, *htb1*, and *cfh4*). Data represent the mean ± s.d. from three biological replicates and *p* values were calculated using a student’s t-test (^***^p < 0.001, ^**^p < 0.01, ^*^p < 0.05, ns: not significant).

To elucidate the molecular basis of chromosome segregation defects under iron-deficient conditions, we analyzed the genome-wide condensin binding profile by ChIP-seq using a Cut14-12Pk–tagged strain cultured under iron-replete (100 µM FeCl_3_) and iron-depleted (250 µM DIP) conditions. Global analysis revealed a widespread reduction in condensin binding under iron-limiting conditions (Fig. 3A), with particularly pronounced loss at discrete genomic loci.

Notably, the top 40 high-occupancy condensin binding peaks showed substantial signal reduction in iron-depleted cells (Fig. 3B). Many of these peaks corresponded to Ace2 and Ams2 target genes, including *adg1*, *adg3*, *hta1*/*htb1*, and *cfh4*, which consistently exhibited reduced condensin enrichment following DIP treatment (Fig. 3C). Metagene analysis revealed reduced condensin occupancy centered at peak summits across high-affinity sites, particularly at Ace2/Ams2-regulated genes (Fig. 3D). In contrast, condensin binding at tRNA, rRNA, and non-coding RNA loci remained largely unaffected by iron depletion (Fig. 3D), suggesting that local condensin loading defect specifically to RNA polymerase II-transcribed genes.

These findings were further validated using ChIP-qPCR, which confirmed significantly decreased condensin binding at *adg1*, *adg3*, *htb1*, and *cfh4* under iron-depleted compared to iron-replete conditions (Fig. 3E). Together, these results demonstrate that iron availability modulates condensin binding, particularly at strong affinity sites associated with Ace2/Ams2-regulated genes.

### Cohesin binding was relatively insensitive to iron depletion

**Fig. 4.**
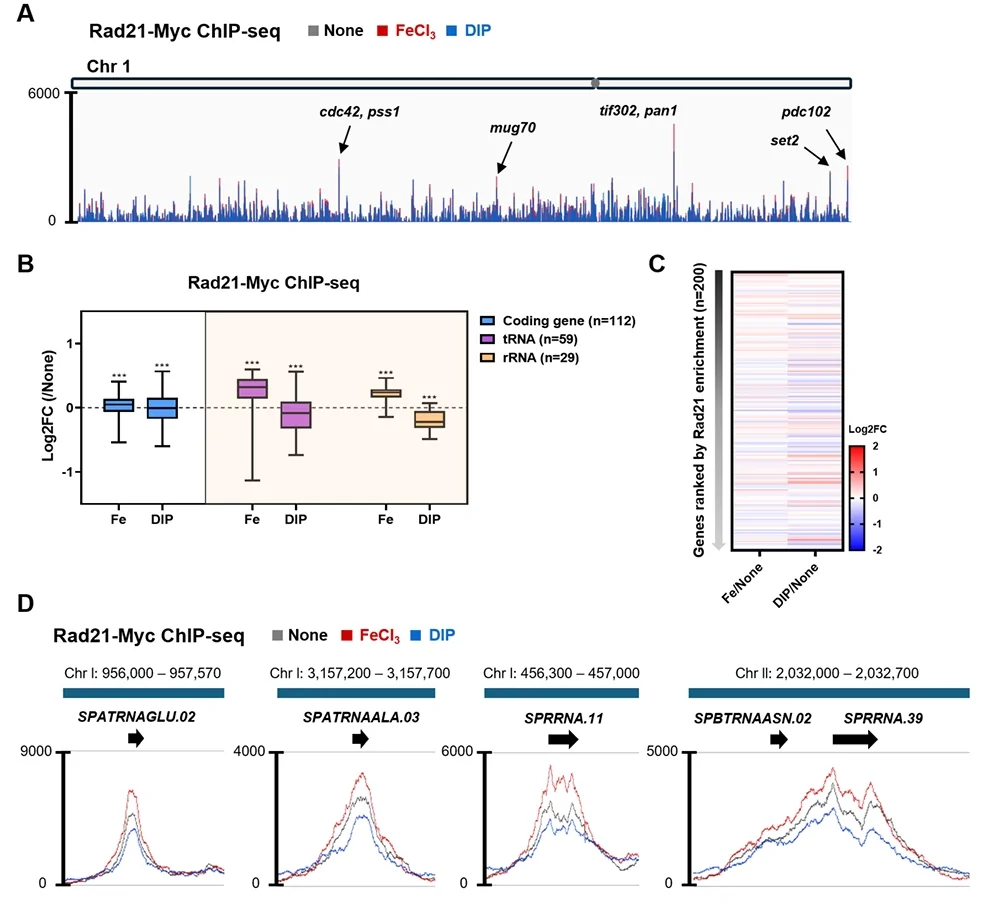
Cohesin binding under iron-replete and iron-depleted conditions. (A) ChIP-seq profile of Rad21-Myc binding on chromosome I under both iron conditions. The top five cohesin-binding genes on chromosome I are shown in black. (B) Box plot showing average Rad21-Myc enrichment across gene classes. *p* values were calculated using one sample t-test (^***^p < 0.001, ^**^p < 0.01, ^*^p < 0.05, ns: not significant). (C) Cohesin binding patterns at the top 200 high-affinity sites under iron-replete and iron-depleted conditions. (D) Rad21-Myc binding at *tRNA* and *rRNA* genes under different iron conditions. Line plots indicate enrichment under each condition. Each snap is enlarged image captured from IGV.

To determine whether the effect of iron depletion on SMC complex function is specific to condensin, we examined cohesin binding under the same conditions by analyzing the Rad21 kleisin subunit of cohesin complex. ChIP-seq was performed using a Rad21-13Myc-tagged strain cultured in iron-replete (100 µM FeCl_3_) and iron-depleted (250 µM DIP) media. In contrast to the pronounced reduction in condensin occupancy observed under iron-deficient conditions, global cohesin binding remained largely unchanged (Fig. 4A).

Metagene analysis confirmed that Rad21 enrichment at RNA polymerase II (RNAP II)-transcribed genes was not significantly altered by iron depletion (Fig. 4B). Examination of the top 200 cohesin binding peaks revealed no consistent or statistically significant global differences between iron-replete and iron-deficient conditions (Fig. 4C). However, modest changes were detected at other genomic loci. Specifically, cohesin occupancy slightly increased on tRNA genes and slightly decreased on rRNA genes under iron-depleted conditions (Fig. 4B and 4D).

Collectively, these findings suggest that cohesin binding is relatively insensitive to iron availability, in contrast to the iron-dependent regulation observed for condensin. This indicates differential regulation of SMC complexes under iron-limiting conditions.

### Expression of SMC complex components was unaffected by iron depletion

**Fig. 5.**
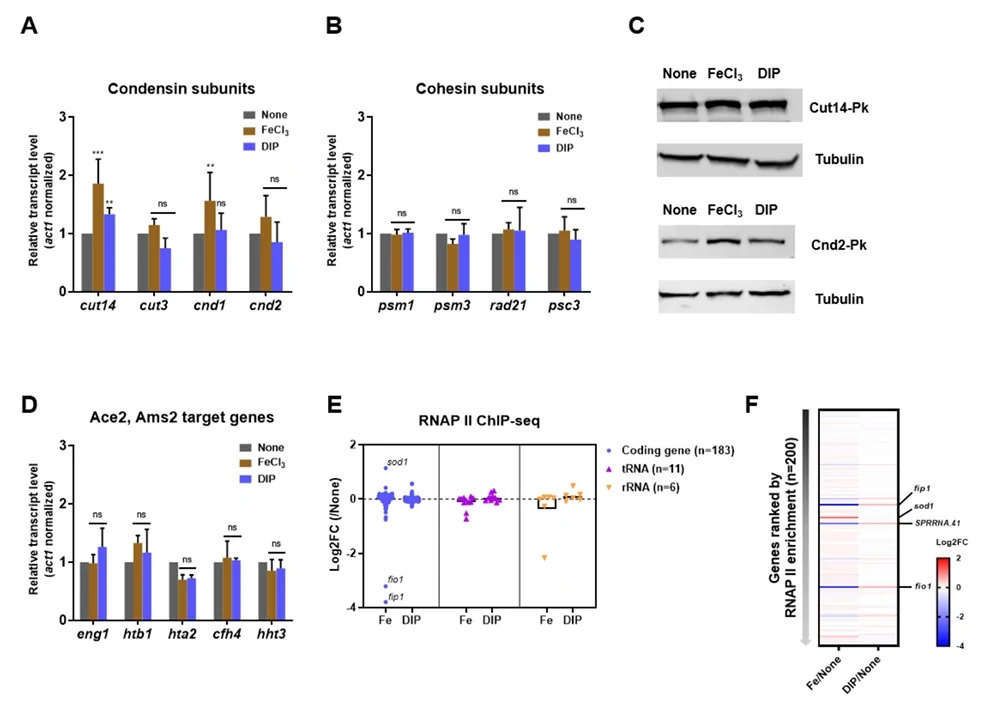
Expression of SMC complex components and Ace2/Ams2 target genes under varying iron conditions. (A, B) Transcript levels of condensin subunits (A) and cohesin subunits (B) under iron-replete (100 µM FeCl_3_) and iron-depleted (250 µM DIP) conditions. Data represent the mean ± s.d. from three biological replicates and *p* values were calculated using student’s t-test (^***^p < 0.001, ^**^p < 0.01, ^*^p < 0.05, ns: not significant). (C) Western blot showing protein levels of Cut14 and Cnd2 under different iron conditions. (D) Transcript levels of Ace2/Ams2 targets (*eng1*, *htb1*, *hta2*, *cfh4*, and *hht3*) under the same conditions. Data represent the mean ± s.d. from three biological replicates and *p* values were calculated using student’s t-test (^***^p < 0.001, ^**^p < 0.01, ^*^p < 0.05, ns: not significant). (E) Scatter with bar plot showing RNA polymerase II enrichment at genes across classes. Each dot on plot indicates individual gene value. (F) RNA polymerase II binding patterns at the top 200 high-affinity sites under iron-replete and iron-depleted conditions.

To investigate whether reduced condensin occupancy under iron-depleted conditions is due to altered expression, we first assessed mRNA levels of condensin subunits. qRT-PCR analysis showed that transcript levels of *cut14*, *cut3*, *cnd1*, and *cnd2* were modestly elevated under iron-replete conditions, particularly *cut14* and *cnd1*, rather than decreased under iron deficiency (Fig. 5A). Transcript levels of cohesin subunits remained stable across both conditions (Fig. 5B). Consistent with these results, western blot analysis revealed no major changes in protein levels of Cut14-Pk or Cnd2-Pk under iron-depleted conditions (Fig. 5C). These findings indicate that iron availability does not significantly influence the transcriptional or translational expression of SMC complexes.

We next assessed whether transcription of condensin-bound target genes was altered. Despite reduced condensin occupancy at Ace2- and Ams2-regulated loci under iron-depleted conditions, transcript levels of these genes remained largely unchanged (Fig. 5D).

Given the potential link between condensin and transcriptional regulation by RNAP II and TATA-binding protein, we evaluated RNAP II occupancy at its top 200 binding sites under varying iron conditions. ChIP-seq analysis showed that RNAP II binding was broadly preserved across coding genes, with the exception of iron-responsive genes, such as *fip1* and *fio1*, which exhibited increased RNAP II enrichment (Fig. 5E and 5F). RNAP II occupancy at tRNA and rRNA genes was also unaffected by iron depletion (Fig. 5E).

These observations suggest that the reduction in condensin binding under iron-deficient conditions occurs independently of major changes in SMC complex abundance or global transcriptional activity.

### Iron deficiency induced localized changes in genome organization

Given the established role of condensin in mitotic chromosome architecture and centromeric clustering, we hypothesized that iron depletion may affect higher-order genome structure through reduced condensin binding. To test this, we performed Hi-C analysis on cells grown in iron-replete (100 µM FeCl_3_) and iron-deficient (250 µM DIP) conditions.

**Fig. 6.**
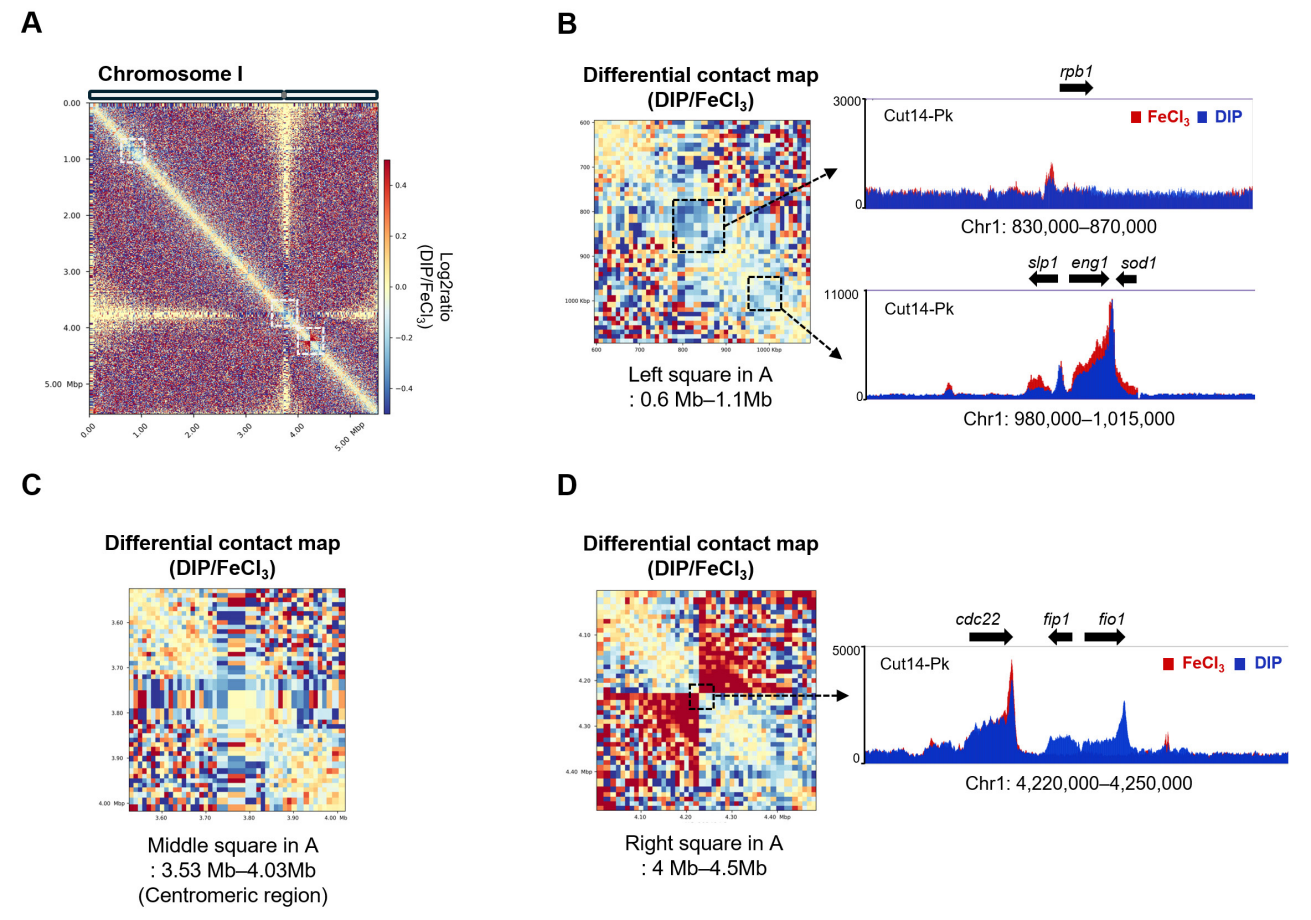
Genome organization under iron-replete and iron-depleted conditions. (A) Hi-C differential contact map (10-kb binning) for chromosome I between iron-replete (100 µM FeCl_3_) and iron-depleted (250 µM DIP) conditions. Regions with altered contacts are outlined with white dashed boxes. (B) Differential contact map and ChIP-seq enrichment profiles of Cut14-Pk (left square in A; chromosome I: 0.6-1.1 Mb). (C) Differential contact map of centromeric region (middle square in A; chromosome I: 3.5–4.0 Mb). (D) Differential contact map and ChIP-seq enrichment of Cut14-Pk for the right square in A (chromosome I: 4.22–4.25 Mb), encompassing iron-uptake genes *fip1* and *fio1*.

Global Hi-C contact maps revealed that overall 3D genome organization was largely preserved under iron-depleted conditions, indicating the absence of widespread chromatin reconfiguration. However, differential contact analysis revealed focal decreases in chromatin interactions at specific genomic regions, including a ~1 Mb domain and centromeric regions (Fig. 6A). Closer inspection of the ~1 Mb region revealed reduced chromatin contacts near the *rpb1* and *eng1* loci, both of which exhibited reduced condensin occupancy following DIP treatment (Fig. 6B). Similar reductions in interaction frequency were observed at centromeres, regions typically enriched for condensin (Fig. 6C). However, condensin binding intensities were not quantified at centromeres due to repetitive nature of centromeric DNA.

Conversely, a region near 4.2 Mb exhibited enhanced chromatin contacts under iron-depleted conditions, correlating with increased condensin binding at nearby iron-regulated genes, including *fio1* and *fip1* (Fig. 6D). These data support that changes in 3D genome architecture reflect locus-specific alterations in condensin occupancy.

Together, our results demonstrate that iron deficiency induces local, but not global, changes in genome organization through modulation of condensin binding. These findings reveal an unanticipated role for iron homeostasis in regulating chromatin architecture.

### Iron depletion disrupted long-range chromosomal associations between centromeric regions and Ace2 target loci

**Fig. 7.**
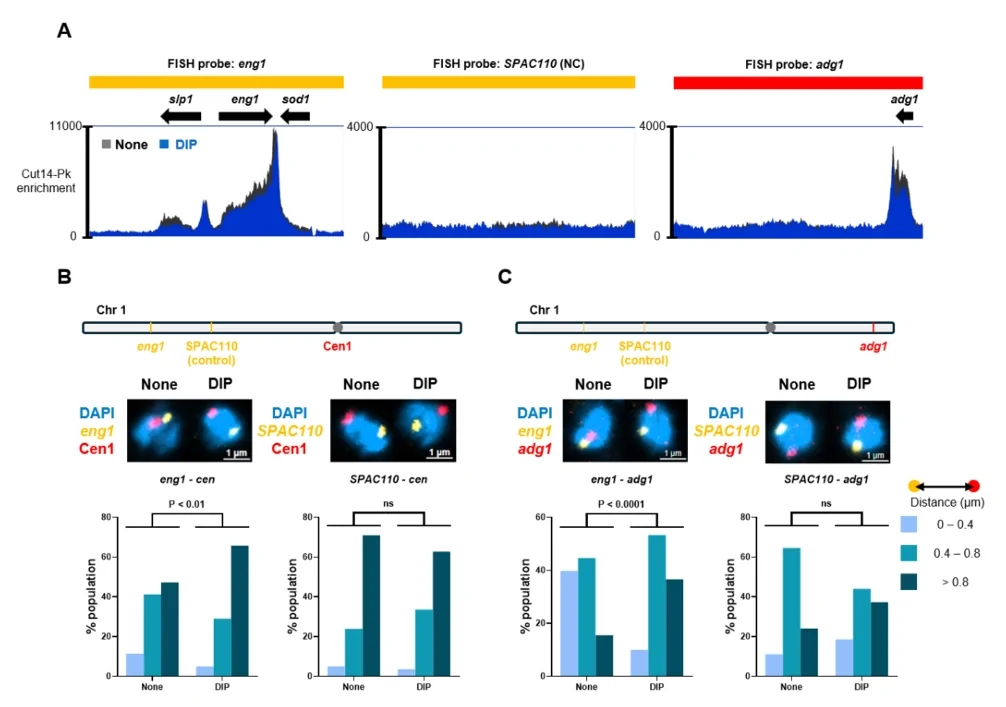
Reduced condensin-mediated chromosomal associations under iron-depleted conditions. (A) Genomic positions of FISH probes targeting Cut14-Pk-enriched loci near *eng1* and *adg1*, and a negative control (*SPAC110*). ChIP-seq signals confirm condensin binding at *eng1* and *adg1* but not at the control locus. (B, C) FISH analysis of inter-locus distances between *eng1* and centromere I (*cnt1*), or *eng1* and *adg1*, under iron-replete and iron-depleted conditions. Representative images are shown above plots. Distances were measured in > 100 cells and binned. Statistical validation was performed based on a Mann-Whitney U test.

Previous ChIA-PET analysis (Kim et al., 2016b) has identified prominent long-range interactions between centromeric regions and distal Ace2 target genes, including *eng1* and *adg1*. In our Hi-C analysis, we observed a local reduction in chromatin contacts at these loci under iron-deficient conditions, suggesting that iron availability influences higher-order genome organization. To examine this effect at the single-cell level, we performed DNA FISH to quantify the spatial proximity between centromere 1 (Cen1) and Ace2 target genes (*eng1*, *adg1*), along with a non-target control locus (*SPAC110*), under normal and iron-depleted (DIP-treated) conditions (Fig. 7A).

Consistent with the Hi-C data, DNA FISH revealed a significant increase in the physical distance between *eng1* and Cen1 under iron depletion (Fig. 7B). In contrast, the distance between Cen1 and *SPAC110* remained unchanged, indicating that the observed disruption was not due to global nuclear reorganization (Fig. 7B). To further assess coordinated spatial changes among Ace2 targets, we measured inter-locus distances between *eng1* and *adg1*. Under iron-deficient conditions, the *eng1*–*adg1* distance increased significantly, whereas distances between *SPAC110* and *adg1* were unaffected (Fig. 7C).

These findings demonstrate that iron deficiency disrupts condensin-dependent long-range chromosomal interactions between centromeres and Ace2-regulated genes. This supports a model in which iron availability modulates 3D genome architecture by influencing condensin-mediated nuclear positioning of key transcriptional targets.

### Zinc deficiency selectively reduced condensin binding at high-affinity genomic loci

To determine whether the reduction in condensin binding observed under iron-deficient conditions reflects a metal-specific response, we examined the effect of zinc depletion on condensin occupancy. ChIP-seq analysis of Cut14-Pk was performed in cells treated with the zinc chelator TPEN at 50 µM and 100 µM.

**Fig. 8.**
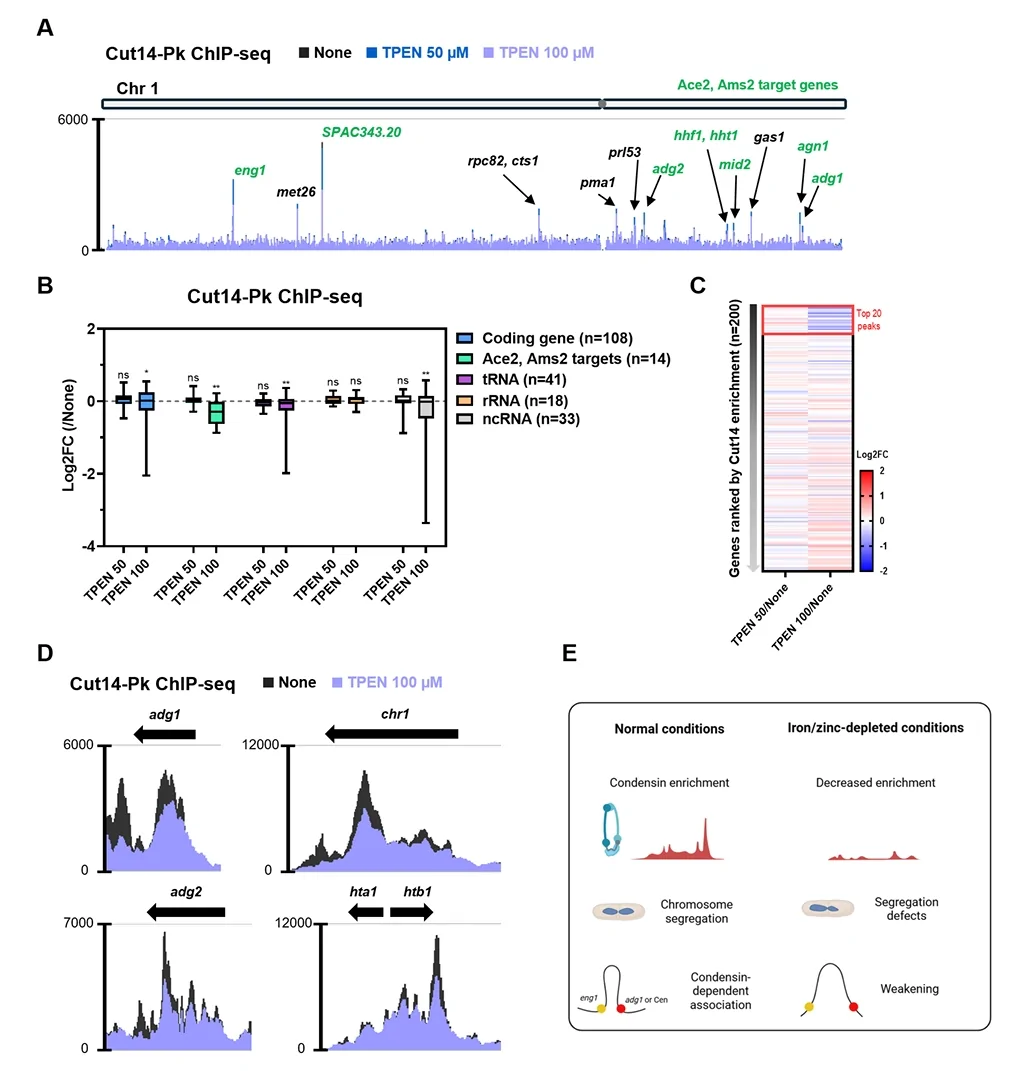
Condensin binding patterns under zinc-depleted conditions. (A) ChIP-seq profiles of Cut14-Pk binding on chromosome I under zinc-depleted conditions (50 µM and 100 µM TPEN). Ace2/Ams2 targets are shown in green. (B) Box plot showing average Cut14-Pk enrichment across gene classes. *p* values were calculated using one sample t-test (^***^p < 0.001, ^**^p < 0.01, ^*^p < 0.05, ns: not significant). (C) Condensin binding profiles at the top 200 high-affinity sites under zinc-depleted conditions. (D) Reduced condensin binding at target genes under zinc depletion (100 µM TPEN). Bar plots show enrichment levels at these loci. Each snap is enlarged image captured from IGV. (E) Graphical abstract of this study. This figure created with BioRender (https://BioRender.com).

Zinc depletion led to a dose-dependent reduction in condensin binding, with pronounced losses observed at specific high-affinity loci, including several Ace2- and Ams2-regulated genes (e.g., *eng1*, *adg2*, *mid2*, and *adg1*) (Fig. 8A). Quantitative classification of condensin enrichment across gene categories revealed that RNA polymerase II-transcribed genes, particularly Ace2 and Ams2 targets, exhibited the greatest loss of condensin occupancy, whereas tRNA and other non-coding RNA loci were largely unaffected (Fig. 8B). While iron deficiency significantly reduced condensin binding across the top 40 strongest peaks (Fig. 3), zinc depletion predominantly affected the top 20 peaks, indicating a more focused yet specific effect (Fig. 8C). ChIP-seq track profiles confirmed this pattern, showing reduced condensin enrichment at representative high-affinity sites following 100 µM TPEN treatment (Fig. 8D).

Together, these results indicate that zinc deficiency, like iron deficiency, impairs condensin binding at high-affinity genomic loci, albeit with a more restricted scope and magnitude. These findings suggest that condensin occupancy, chromosomal segregation, and condensin-mediated chromatin structure are modulated by the intracellular availability of both iron and zinc (Fig. 8E). Future studies will be required to determine whether similar regulatory mechanisms apply to other biologically relevant metal ions involved in chromatin organization.

## Discussion

Our findings demonstrated that iron deficiency compromised condensin-mediated chromosomal associations at specific genomic loci, revealing a previously unrecognized role for iron/zinc homeostasis in maintaining three-dimensional genome architecture. Hi-C analysis indicated that, although global chromatin organization remained largely intact under iron-depleted conditions, specific long-range interactions were selectively weakened. In particular, chromatin contacts between centromeric regions and Ace2-regulated genes, such as *eng1* and *adg1*, were diminished, consistent with a localized disruption of higher-order chromatin structure. To validate these Hi-C observations, we employed DNA FISH targeting previously characterized long-range interactions between *cen1*–*eng1* and *eng1*–*adg1* (Kim et al., 2016b), which are stabilized by condensin. Under iron-depleted conditions, spatial proximity between these loci was significantly reduced, confirming the disruption of condensin-dependent chromosomal associations at the single-cell level. These results support that iron availability is essential not only for maintaining condensin binding at individual loci but also for preserving long-range chromatin interactions critical for nuclear organization.

Although condensin transcript and protein levels remained unchanged under iron and zinc depletion, binding affinity was selectively reduced at high-occupancy sites. This suggests the existence of a regulatory mechanism that modulates condensin activity post-translationally or via the chromatin context. In contrast, cohesin binding at protein-coding genes was largely unaffected under iron and zinc depletion, underscoring the specificity of the condensin response. Minor changes were observed at non-coding RNA loci: cohesin occupancy at tRNA genes modestly increased under iron-replete conditions and decreased under depletion, while rRNA loci exhibited the opposite trend. Elucidating the molecular mechanisms by which metal deficiency impairs condensin function remains an important direction for future research.

In our earlier work, we observed defects in condensin binding activity and chromosome segregation under iron-depleted conditions. To determine whether this defect is a specific response to iron deficiency or a more general response to metal depletion, we further examined the effect of zinc. Interestingly, zinc deficiency also led to a significant reduction in condensin occupancy at its target sites. However, the current findings are insufficient to generalize the role of metal ions broadly. Further studies are needed to assess the impact of other essential biological metals, such as magnesium, copper, manganese, nickel, and others. Divalent metal ions play essentials in genome maintenance. Previous studies have demonstrated that iron deficiency can alter histone modifications (Gallagher et al., 2018), while zinc deficiency has been shown to reduce chromatin accessibility (Ocampo et al., 2024). Moreover, zinc deficiency induces replication stress and dysregulate the expression of DNA damage response genes (Holtzen et al., 2024; Yan et al., 2008). In addition to iron and zinc, magnesium functions as a critical cofactor for DNA topoisomerase, thereby contributing to genomic stability (Sissi and Palumbo, 2009). Magnesium deficiency has also been associated with accelerated cellular senescence through activation of DNA damage response pathways (Killilea and Ames, 2008). However, the broader impact of essential metals on chromosomal architecture and other nuclear processes remains largely unexplored.

Collectively, our results identify condensin as a chromatin regulator whose activity is sensitive to intracellular metal ion status, particularly iron and zinc. This metal-dependent regulation may constitute a broader mechanism by which cells integrate metabolic cues into chromatin architecture to safeguard genome stability under nutrient-limiting conditions. Although this study was conducted in *S. pombe*, the observed impairments in condensin binding and chromosome segregation under iron- and zinc-deficient conditions have potential implications for higher eukaryotes. Given the evolutionary conservation of SMC complexes, it is plausible that the insufficient availability of essential metal ions in mammalian cells similarly disrupts condensin function, leading to mitotic defects and genomic instability. Future studies in multicellular systems will be necessary to determine whether metal homeostasis represents a general regulatory axis for chromosome segregation fidelity and nuclear organization across eukaryotes.

## Supplementary Information

Table S1. Strain list of *S. pombe*
