# Supplementary material for: Metal ion homeostasis regulates condensin-dependent chromatin architecture and chromosome segregation in Schizosaccharomyces pombe
Source: J Microbiol. 2025 Aug 29;63(9):jm.2505008. doi: 10.71150/jm.2505008 (PMC13577065; doi:10.71150/jm.2505008)
Supplement: Supplementary file 1 [file jm-2505008-Supplementary-Table-S1.pdf]

Table S1. Strain list of *S. pombe*

Strains	Additional	Experiment
**SPK425**	Wild type	Hi-C, RT-PCR
**FY14415**	*his2 ade6-M210 / ade6-M216 ch16*	Mini chr loss assay
**FY9897**	*cut14-208*	RT-PCR
**SPO285**	*cut14-12Pk*	ChIP, WB
**SPO411**	*rad21-13Myc<<KanMX*	ChIP
**SPJH010**	*mat1M17 lys1-131 ura4+::Padh1-OsTIR1-F74A, cut14-3sAID*	RT-PCR
**SPSH001**	*mat1MΔ17 lys1-131 ura4+::Padh1-OsTIR1-F74A, cut3-3sAID*	RT-PCR
**SPSH003**	*cnd2-12pk-KanMX*	WB
